# An atypical case of hepatic cavernous hemangioma

**DOI:** 10.1186/1757-1626-2-181

**Published:** 2009-11-02

**Authors:** Alfio Brogna, Rosario Ferrara, Anna Maria Bucceri, Carla Virgilio, Giuseppe Scalisi

**Affiliations:** 1 Department of Internal Medicine. Gastroenterology Unit. S. Luigi Hospital. Viale Fleming 24, 95100, Catania, Italy

## Abstract

**Introduction:**

The case of an atypical hepatic angiocavernoma is referred. The lesion, first described as a hypoechogenic area compared to the surrounding parenchyma, with anechogenic shoots inside, suggestive for vascular structures developed one year later into a totally asonic area with frayed margins. This change is very unusual and uncommon for this kind of lesions.

**Case presentation:**

The case of a 74-year old caucasian male, complaining of slight dyspeptic symptoms (post-prandial fullness and bloating) is referred. The routine blood tests were all normal. Abdominal ultrasound showed a large, roughly round-shaped lesion (diameter 14 cm) in the VIII hepatic segment diagnosed as hepatic angiocavernoma, which turned unexpectedly in a cystic like lesion one year later.

**Conclusion:**

The atypical angioma's degeneration could account for one of the causes of the patient's exitus. It could be related to blood seizure by the large hepatic angioma due to the intratumoural haemorrhage.

## Introduction

The angioma represents the liver's most frequent benign tumour, with an approximate incidence of 4% on population. Angiomas can hit any age bracket, but they are more frequent in adults, especially in women (ratio M/F = 1/4-6). Most of angiomas are small, asymptomatic and detected by chance. Large angiomas can appear as palpable abdominal masses, with pain at the right hypochondrium and a bloated loins feeling due to capsular pressure, haemorrhage or thrombus; those ones localised in the sub-capsular area are subject to easy breaking, with subsequent intraperitoneal haemorrhage; the thrombocytopenia caused by the seizure and destruction of platelets within the large cavernous angioma (syndrome of Kasabach-Merritt) is another complication [1]. It occasionally occurs in children and rarely in adults. Angiomas detected in adults generally reach a stable size and their modification in aspect and size is uncommon [2,3]. Angiomas can increase their size either during pregnancy or after the administration of estrogens, suggesting the tumour depends on hormones.

Histologically, the angioma constists of a vascular proliferation where the vessels are separated by a delicate fibrous stroma. It is possible to find thrombi within the vascular lacunae. The angioma is provided with a fibrous capsule, which at times can appear to be calcific [1].

Its typical sonographic aspect is that of a small roundish area, with hyperechoic definite lips. Most angiomas have a diameter smaller than 3 cm. Circa 30% of angiomas show a hypoechoic, isoechoic or mixed structure, only seldom appearing clearly cystic [1].

## Case presentation

**Figure 1:**
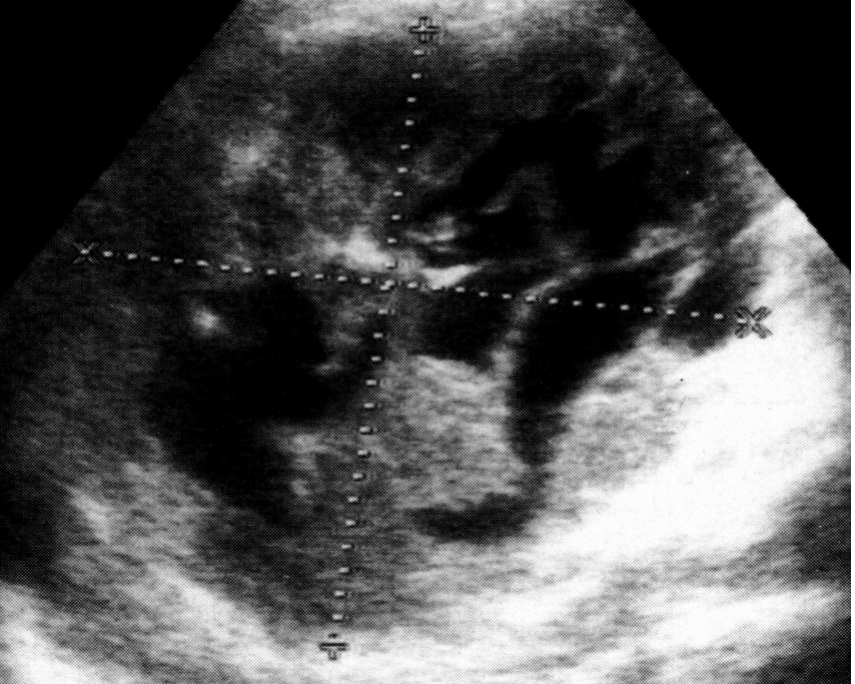
**Hepatic hemangioma (first presentation)**. Large, roughly roundish formation, located in the VIII hepatic segment, 14 cm in diameter, basically hypoechoic compared to the surrounding parenchyma. It was chacterised by several anechoic shoots with a serpentine-like course, very suggestive of vascular structures.

A 74 year-old caucasian male complained of light dyspeptic symptoms, characterized by post-prandial repletion and fullness. Moreover, the patient showed a light cardio-respiratory insufficiency. The routine blood parameters were all normal. Abdominal ultrasonography showed the presence of a large, roughly roundish formation, located in the VIII hepatic segment, 14 cm in diameter, basically hypoechoic compared to the surrounding parenchyma. It was chacterised by several anechoic shoots with a serpentine-like course, very suggestive of vascular structures (Figure 1). At first, this formation was considered to be an atypical cavernous haemangioma. Due to the peculiar appearance of such a lesion, it was deemed necessary to perform an echo colour-Doppler and a CT, even though no risk factor for malignant lesion was suspected. The diagnosis of an cavernous haemangioma was confirmed. The patient was proposed a planning of sonographic follow-up (every six months at first), in consideration of angioma's remarkable size, order to monitor any possible change.

**Figure 2:**
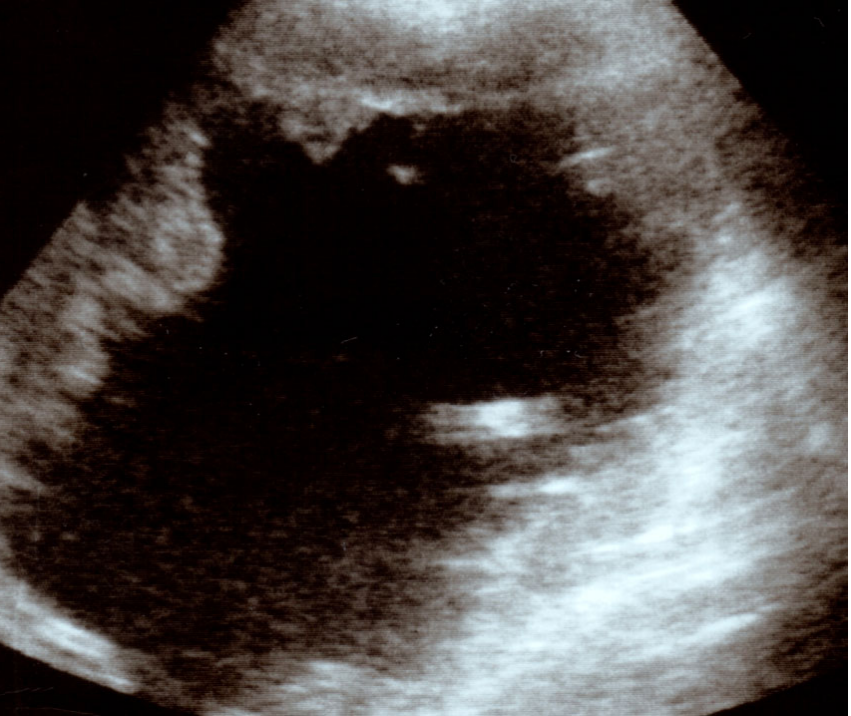
**Development of hepatic hemangioma**. Voluminous anechoic formation (axis longer than 20 cm), with non-definite and frayed lips, occupying a large portion of the right lobe.

One year after the angioma had been detected, abdominal ultrasonography showed a deep alteration of the lesion, which appeared as a more voluminous anechoic formation (axis longer than 20 cm), with non-definite and frayed lips, occupying a large portion of the right lobe (Figure 2). The colour-Doppler did not report any blood flow within the injury. The patient's circulatory conditions showed a decline which led him to death shortly afterwards.

## Conclusion

In our case the observed mass was totally different from that of a typical cavernous haemangioma, characterised by a mixed, complex structure, whose solid component is intermingled with hypo-anechoic haematic lacunae. Such lacunae are commonly disomogeneous and with irregular margins, roughly roundish or polygonal [4], and not vessel-like as the lesion we detected.

Ultrasonographic examination performed one year later showed that the angioma had developed and changed its echostructure. The original lesion come out to have turned into a large anechoic formation with irregular and frayed lips.

Many authors support that hemangiomas are congenital benign hamarthomas while others have suggested they might originate from hepatic areas of focal necrosis and regeneration. They can grow bigger in the early phases of life due to dilatations of the pre-existing vessels rather than to the development of new vessels, but tend to remain stable as for dimensions in adulthood, especially those ones smaller than 4 cm. Nevertheless, if angiomas get bigger, they can undergo several changes, such as: internal hemorrhage with necrosis, thrombosis, myxomatous modifications, fibrosis and calcifications. Such modifications could account for the different echo-pattern [5], but generally they do not involve the whole lesion, like in our observed case.

Some reports have documented hemangiomas growing bigger in time. A spontaneous grow of hemangiomas has been suggested and also other causes have been envisaged, for instance a chronic therapy with estrogens[6]. In our case, we can exclude a history of therapy whit drugs accounting for the growth of the formation. Moreover the particular modification of the lesional echo-pattern, which had become totally anechoic, would firstly incline for an intralesional hemorrhage [6-8] with a possible fragmentation of vascular structures and consequent transformation into a wholly liquid formation.

Secondarily, the anechoic formation, detected in the last sonographic examination, could be related to the angioma's cystic degeneration [9,10].

The hepatic cavernous hemangioma with cyst formation is very rare. In literature only three cases of liver's cystic hemangioma have been reported. Two of them have stated the formation of multilocular cysts, whereas the other one has shown a single unilocular cyst. Tumour's cystic degenerations have been attributed to necrotic changes caused by the development of an infarction area due to vessels' occlusion [9].

In our case, considered the ragged shape of the formation detected within the angiocavernoma, the tumour's cystic degeneration seems rather unlikely. In this case the anechoic formation should have been roundish with regular walls.

In time, the patient's hemodynamic conditions, well balanced at first, started to worsen. This could be related to blood seizure by the large hepatic angioma due to the intratumoural haemorrhage. In spite of the presence of this gigantic angioma, signs and symptoms typical of the Kasabach-Merritt syndrome were not reported.

In order to better study the transformation of the angioma detected in the second ultrasound scanning and indentify a possible relation with the worsening of the patient's cardiac conditions, it would have been necessary to carry out further tests, which couldn't be performed due to the patient's exitus.

In any case, we consider this case very interesting, both for the angioma's initial echografic appearance and for its particular and uncommon transformation which probably could be responsible for the worsening of hemodynamic condition that caused the patient's exitus.

We maintain that our case represents an interesting communication first for the early aspect of the cavernous haemangioma, that is quite different from other hepatic cavernous hemangiomas reported in the literature. Moreover its interest can be attributed to the peculiar change of the lesion that turned into a liquid concistency. Such a change is rather uncommon, as it involves the whole lesion.

We underline that our data result from the ultrasonographic monitoring of the lesion which has been judged, in any way, along with the patient's clinical conditions. We think that the results of our case report may stimulate to closely monitorate all those angiomatous hepatic lesions whose ultrasonographic appearance is closely related to the one we are presenting.

## Abbreviations

CT: computed tomography.

## Competing interests

The authors declare that they have no competing interests.

## Authors' contributions

AMB, GS and CV collected the data and drafted the manuscript. AB and RF took care of the patient during hospitalization. All authors read and approved the final manuscript.

## Consent

Written informed consent was obtained from the patient for publication of this case report and accompanying images. A copy of the written consent is available for review by the Editor-in-Chief of this journal.
